# Neurosurgery as an immune anchor point: a translational framework for perioperative immunoengineering

**DOI:** 10.3389/fimmu.2026.1808211

**Published:** 2026-04-17

**Authors:** Zheng Hu, Jun Luo, Jianyun Lou, Juntao Deng, Zi tao Gong, Jinming Chen

**Affiliations:** 1 The First Affiliated Hospital of Gannan Medical University, Ganzhou, Jiangxi, China; 2 Department of Neurosurgery, the First Affiliated Hospital of Gannan Medical University, Ganzhou, Jiangxi, China; 3 Gannan Medical University, Ganzhou, Jiangxi, China

**Keywords:** Neuroimmunology, meningeal lymphatics, glymphatic system, blood–brain barrier, microglia, immunotherapy, perioperative immunity, glioblastoma

## Abstract

Neurosurgical diseases—including brain tumors, hemorrhage/trauma, ischemia, infection, epilepsy, and spinal cord injury—share convergent neuro-immune mechanisms. In the acute phase, sterile inflammation and barrier disruption trigger innate immune cascades. During the subacute phase, immune resolution and clearance determine the quality of tissue repair. In the chronic phase, persistent immune-glia interactions and synaptic remodeling influence epileptogenesis and long-term cognitive outcomes. Recent discoveries—such as the meningeal immune niche, meningeal lymphatic system, and glymphatic clearance pathways—have redefined the classical concept of “CNS immune privilege.” The central nervous system is no longer viewed as immune-isolated, but rather as a compartment whose immunity can be directly modulated by surgical intervention and perioperative management.

This review proposes a conceptual framework in which neurosurgery serves as a programmable “immune anchor point.” By integrating knowledge of neuro-immune interface architecture and temporal dynamics, we establish a closed-loop model encompassing structural pathways, immune dynamics, delivery/timing, and efficacy/toxicity. This paradigm shift aims to accelerate breakthroughs in CNS immunotherapies. The article unfolds along three main themes: (1) the structural foundations of neuro-immune communication—including barrier systems, the meningeal immune niche, and meningeal lymphatic–glymphatic coupling; (2) temporal immune dynamics across acute, subacute, and chronic phases, and their roles in edema, secondary injury, and failed resolution; and (3) the brain tumor immune microenvironment, with a focus on surgical synergy and analysis of why immunotherapies (checkpoint inhibitors, vaccines, oncolytic viruses, cell therapies) have largely failed in glioblastoma. Finally, we propose a translational roadmap integrating perioperative immune management, spatial omics stratification, and local immunoengineering.

## 1 Introduction

Traditional neurosurgical narratives have centered on structural variables such as “mass effect, hemorrhage load, ischemic perfusion, intracranial pressure/brain edema, and functional area preservation”-factors that indeed determine initial injury and direct surgical benefit. However, growing clinical observations indicate that patient outcomes can diverge significantly even with similar imaging and surgical parameters. For instance, intracerebral hemorrhages of comparable volume may follow starkly different edema trajectories, with some progressing rapidly toward malignant intracranial hypertension while others remain relatively stable (1). Similarly, glioblastoma (GBM) patients undergoing maximal safe resection exhibit highly heterogeneous recurrence patterns and responses to adjuvant therapies (2). In aneurysmal subarachnoid hemorrhage (SAH), the incidence of delayed cerebral ischemia and long-term cognitive and emotional sequelae varies widely (3). Such variability often cannot be explained solely by “structural damage” but is closely linked to the intensity, temporal rhythm, spatial distribution, and resolution capacity of immune responses.

The historical concept of “CNS immune privilege” emphasized that the blood–brain barrier (BBB) restricts immune cell and antibody entry, thereby isolating the CNS from peripheral immunity (4). However, over the past decade, key discoveries have reframed this understanding: the meningeal immune niche is not a sparse “border zone” but a rich and dynamic immune tissue (5); meningeal lymphatic vessels establish a traceable drainage and antigen exchange route between the CNS and peripheral immune system (6); the glymphatic system—linked to CSF dynamics, sleep, and astrocytic aquaporin-4 polarity—governs the clearance of inflammatory mediators, metabolic waste, and protein aggregates (7, 8); and a”local supply” pathway may exist for myeloid cell mobilization between skull bone marrow and meninges (9). Thus, CNS immunity is no longer a simple binary of “access vs. no access” but a dynamic system defined by when, where, and how immune components enter and persist; which cells they interact with; and whether they can transition timely into resolution and repair.

For neurosurgery, this paradigm shift offers two key implications. First, it transforms many clinical variables from “uncontrollable heterogeneity” into monitorable, stratifiable, and intervenable parameters. Examples include: perioperative steroids and stress altering the anti-tumor immune landscape; drainage/shunting and positioning influencing inflammatory clearance; and local delivery into the surgical cavity bypassing the BBB to enhance regional immunotherapy intensity (10–12). Second, neurosurgery possesses unique translational advantages: (i)sampling access-multi-regional tumor tissue, CSF, surgical cavity exudate, and peripheral blood enable”within-subject tripartite validation” (13); (ii)delivery access-intra/postoperative cavity, intrathecal, and intraventricular routes allow local high-concentration exposure and controlled release (14); and (iii)temporal precision-surgery serves as a well-defined”biological anchor point,”facilitating before–after comparisons and dynamic follow-up in immune intervention studies (15).

Under this new paradigm, this review aims to systematically address two core immunological questions:

1. What are the key anatomical portals (barrier-meninges-lymphatic/glymphatic) and temporal dynamics (acute peak control, subacute resolution, and chronic plasticity limitation) of immune responses in neurosurgery-related diseases?
2. How can we leverage neurosurgery’s unique strengths in sampling, local delivery, and perioperative management to translate these principles into clinical strategies that enhance immunotherapy efficacy while maintaining controllable toxicity?

Building on these questions, we propose a “surgery as an immune anchor point” translational framework. This framework extends beyond the mere recognition that surgery can modulate immunity; rather, it integrates three interconnected dimensions into a programmable, closed-loop system. First, it reframes the surgical event as a temporal and biological reference point, enabling pre-operative immune stratification, intra-operative spatial mapping, and post-operative dynamic monitoring within a standardized time axis. Second, it leverages neurosurgeon-accessible portals—the BBB, meningeal niche, and lymphatic/glymphatic pathways—as both therapeutic targets and delivery routes, explicitly linking anatomical structures to modifiable surgical variables (e.g., dural opening, CSF drainage, cavity delivery). Third, it anchors a verifiable clinical translation pathway through a “minimum dataset” (**Table 1**) that integrates tissue-CSF-blood triangulation, perioperative covariate control, and spatially informed local immunoengineering. Thus, the surgical event becomes not merely a structural intervention but a central architect of the patient’s post-injury or post-resection immune landscape.

**Table 1:** Minimum dataset and key covariates for perioperative immune research.

Module	Minimum required variables (Essential)	Recommended time points (Anchored to surgery)
Perioperative Key Covariates (Must Record)	Corticosteroids: Start/daily dose/cumulative/stop. Infection/Antibiotics: Infection (culture/clinical) Y/N + start/end. Drainage/Shunt/Cisternal Opening: Type + duration + volume/setting. Anesthesia/Analgesia/Sedation: Protocol + sedation depth/duration + main analgesic. Surgical Info: Type/scope + key intraoperative events (blood loss/complications).	Continuous perioperative record (summarized for 0–72h, 3–7d post-op).
Peripheral Blood (Systemic Context)	CBC differential (Neut/Lymph/Mono); NLR; CRP (or IL-6, choose one).	T0: Pre-op; T1: Post-op 3–7d; T2: Post-op 2–4w (Optional: 24–72h post-op).
CSF (CNS Dynamics)	Cell count/differential; Protein/Glucose; (Optional: IL-6); If drained: daily volume.	T0: If obtainable; T1: Post-op 3–7d; T2: Post-op 2–4w (Optional: 24–72h post-op).
Tissue/Surgical Cavity (Spatial Niche)	Multi-region sampling (core/edge/perivascular, etc.) when possible. Basic IHC: CD3/CD8, CD68/Iba1; (Optional: PD-L1/Spatial Omics).	Primarily intraoperative; recurrence/second surgery for longitudinal comparison.
Minimum Endpoints (At least one each)	Safety: Edema/seizures/infection/immune-related neurotoxicity. Mechanistic: T0–T2 biomarker changes (Blood+CSF ± Tissue). Clinical: Neurological function/cognition (acute) or PFS/OS (tumor).	Hospital stay + T2 follow-up (tumor: per routine follow-up).

**Table 1** summarizes the minimum dataset and key covariates for perioperative immune research using the “surgical event” as an anchor point. This framework aims to improve comparability across studies and enable pre-specified stratification or confounding adjustment in statistical analysis. T0, Preoperative baseline; T1, Early postoperative (3–7 days); T2, Mid-postoperative (2–4 weeks). Peripheral blood characterizes systemic immune context (e.g., NLR, CRP/IL-6), CSF characterizes CNS immune dynamics and clearance-related changes, and intraoperative tissue/cavity samples capture spatial heterogeneity and niche features. Items marked “Optional/Choose” are enhancements for when resources allow (e.g., PD-L1, spatial omics).

This article follows this closed-loop logic to comprehensively review neuro-immune pathways relevant to neurosurgery and their translational strategies, concluding with a forward-looking roadmap for future research and clinical trials.

## 2 Methods

This narrative review integrates evidence through a translational framework organized around the hypothesis of a closed-loop system: “structural pathways, immune dynamics, delivery/timing, and efficacy/toxicity”. The surgical event serves as the anchor point for organizing time windows and clinically actionable variables (perioperative management, local delivery, sampling, and biomarker stratification).

We searched PubMed/MEDLINE, Web of Science, and Google Scholar for English-language articles published between January 2010 and January 2026, supplemented by backward citation tracking of key reviews and guidelines. Search terms included combinations of: meningeal immunity, meningeal lymphatics, glymphatic system, cerebrospinal fluid dynamics, blood-brain barrier, choroid plexus, cross-referenced with neurosurgery or perioperative.

Inclusion criteria encompassed mechanistic studies, animal research, clinical cohorts, trials, and authoritative guidelines/consensus statements related to: barrier-meningeal-lymphatic/glymphatic clearance pathways; innate/adaptive immune dynamics; perioperative immune modulation; or immunotherapy delivery strategies in neurosurgery-relevant diseases (brain tumors, hemorrhage/trauma, ischemia, infection, epilepsy, spinal cord injury).

Exclusion criteria were: low thematic relevance; purely descriptive reports lacking mechanistic or translational insights; or non-traceable evidence sources.

The initial database search yielded approximately 3,204 records. After removal of duplicates, 1,820 articles were screened based on title and abstract, of which 450 were selected for full-text review. Following full-text assessment against the inclusion/exclusion criteria, 116 articles met the criteria for inclusion. An additional 14 articles were identified through backward citation tracking of key reviews and guidelines, resulting in a total of 130 cited references in this manuscript.

Evidence synthesis followed a “stratified evidence” approach: findings are categorized as (i) mechanistic/animal evidence, (ii) human associative evidence, or (iii) prospective clinical trial evidence. Where conclusions rely primarily on preclinical data with insufficient human causal evidence, we explicitly note research gaps and propose testable hypotheses to inform the design of future perioperative immune management and local immunoengineering trials.

## 3 Structural foundations of the neuro-immune interface: barriers, meninges, and clearance pathways

### 3.1 BBB/BCSFB: from a “wall” to a “tunable valve”

The BBB is a multicellular structure comprising brain microvascular endothelial cells, pericytes, the basement membrane, and astrocytic end-feet. Its core functions are maintaining ionic homeostasis, excluding toxic substances, and limiting unregulated immune infiltration (16). The blood–cerebrospinal fluid barrier (BCSFB), primarily at the choroid plexus epithelium, participates in CSF production and selective immune molecule transport (17).

Neurosurgical pathologies differentially alter barrier properties: tumor neovasculature often exhibits structural anomalies and increased permeability; hemorrhage and trauma disrupt tight junctions via blood degradation products, mechanical strain, and inflammatory mediators; ischemia-reperfusion induces endothelial oxidative stress, leukocyte adhesion, and microthrombosis; and radiotherapy can cause chronic endothelial injury and lasting vascular permeability changes (18–20). Consequently, the same immunotherapeutic or anti-inflammatory strategy may exhibit vastly different “entry-efficacy-toxicity” relationships across disease stages.

#### 3.1.1 Clinically, barrier status critically influences three issues

1. Drug penetration and spatial coverage: The effective concentration of systemically administered immune checkpoint inhibitors (ICIs), antibody drugs, or cytokines reaching tumor-infiltrating margins, perivascular niches, and postoperative residual micro-foci often dictates the therapeutic ceiling (21, 22).
2. Edema and neurotoxicity risk: Barrier disruption does not equate to “easier immunotherapy success.” It can amplify the influx of peripheral inflammatory cytokines into the CNS, exacerbating cerebral edema, lowering seizure thresholds, and even causing immune-related neurotoxicity (23, 24).
3. Immunological implications of perioperative management: Strategies for fluid management, ventilation, osmotic therapy, and analgesia/sedation, while superficially targeting intracranial pressure, fundamentally modulate endothelial inflammation, microcirculation, and immune cell adhesion/migration (10, 25).

Therefore, in research and writing, the BBB should not be viewed merely as an “entry barrier” but as a measurable biological state. When, to what extent, and with what regional heterogeneity it opens, and how quickly it recovers postoperatively, will define the window and safety margin for immune interventions. Incorporating barrier status into patient stratification is a crucial step toward reproducible neurosurgical immuno-translation.

### 3.2 The meningeal immune niche: the CNS’s “sentry post”

Long considered merely a mechanical protector, the meninges are now recognized as immunologically significant. Under homeostasis and in various pathologies, the meninges harbor a rich and dynamic population of innate and adaptive immune cells (composition varies by brain region and state), including macrophages, dendritic cells, neutrophils, T/B cells, and mast cells. These interact with CSF circulation, venous sinus structures, and skull-associated tissues to form a unique immune niche (5, 9).This “sentry post” function is twofold: (i) sensing internal CNS changes (e.g., damage-associated molecular patterns (DAMPs), protein aggregates, tumor antigens), and (ii) communicating with the peripheral immune system via lymphatic drainage, antigen presentation, and cell migration (26, 27).

For neurosurgery, meningeal immunity has direct operative relevance. Dural opening, meningeal handling, skull base procedures, and interventions near venous sinuses can affect local immune cell distribution and activation states (28); Postoperative dural repair materials and adhesion processes may alter the duration of local inflammation and CSF absorption (29); In contexts like infection, arachnoiditis, or radiation-induced changes, meningeal immune responses are prone to chronicity, correlating with persistent headaches, hydrocephalus, recurrent fevers, or fluctuating CSF parameters (30). Importantly, meningeal immunity exhibits spatial heterogeneity. Immune composition and cellular interaction networks likely differ across brain regions, venous sinuses, and dural layers, challenging the traditional assumption that “a single sample represents the whole brain.” This heterogeneity provides a rationale for future region-specific delivery and stratified sampling strategies (31).

While the meninges are increasingly recognized as an immunologically active site, the extent to which meningeal immune responses directly contribute to parenchymal pathology—as opposed to serving as a parallel compartment—remains incompletely understood (32). Furthermore, most studies have been conducted in rodent models, and the translation of these findings to human neurosurgical patients requires cautious interpretation (33).

### 3.3 Clearance determines outcome: how meningeal lymphatic–glymphatic pathways can drag acute inflammation into chronic disease

This section emphasizes an often-underestimated immunological fact: clearance pathways are not mere “background physiology” but a critical valve determining whether inflammation becomes chronic and whether post-injury immunity can successfully transition to resolution. Neurosurgical alterations of CSF dynamics are equivalent to adjusting this valve’s opening.

The glymphatic system describes the process where CSF enters the brain parenchyma along perivascular spaces, exchanges with interstitial fluid, and facilitates the outward clearance of metabolic waste. Meningeal lymphatic vessels drain meningeal and brain surface components to deep cervical lymph nodes and other peripheral lymphoid structures (34, 35). Together, they form the CNS’s core pathway for clearing waste and inflammatory mediators. Their function is thought to be tightly coupled with sleep, arterial pulsatility, astrocytic aquaporin-4 (AQP4) polarity, body position, and CSF dynamics, based largely on preclinical studies (7).

This system is directly modifiable by neurosurgical practice: drainage/shunting, decompressive craniectomy, cisternal opening extent, postoperative positioning, and sedation depth can all influence clearance efficiency. Clinically, delayed postoperative recovery, long-term fatigue and cognitive issues after SAH, and symptom “tailing” in chronic phases may be linked to inefficient clearance of inflammatory mediators or blood degradation products (36, 37). Framing “clearance” within the immune context organizes scattered phenomena into a more testable causal chain:

Impaired Clearance → Chronic Neuroinflammation → Altered Network Plasticity/Cognitive Decline (proposed mechanism).

The implications of impaired clearance may extend beyond “inflammatory mediator retention.” Some studies suggest reduced clearance function may decrease the efficiency of metabolic waste and abnormal protein (e.g., Aβ, tau) efflux, potentially correlating with increased pathological burden and participating in a self-sustaining loop: “impaired clearance, chronic neuroinflammation, and altered network plasticity or cognitive decline (35, 38)”. It must be noted that this chain is better supported by animal and mechanistic studies; direct evidence in humans remains largely indirect, relying on contrast-enhanced MRI studies with small sample sizes and variable methodology, and causal validation requires longitudinal cohorts with standardized imaging biomarkers (39). For neurosurgery, its value lies in proposing a testable hypothesis: Taken together, these observations support the testable hypothesis that optimizing CSF dynamics and clearance efficiency may simultaneously influence acute inflammation control and long-term outcome trajectories—a premise that warrants investigation in well-designed prospective cohorts.

**Figure 1:**
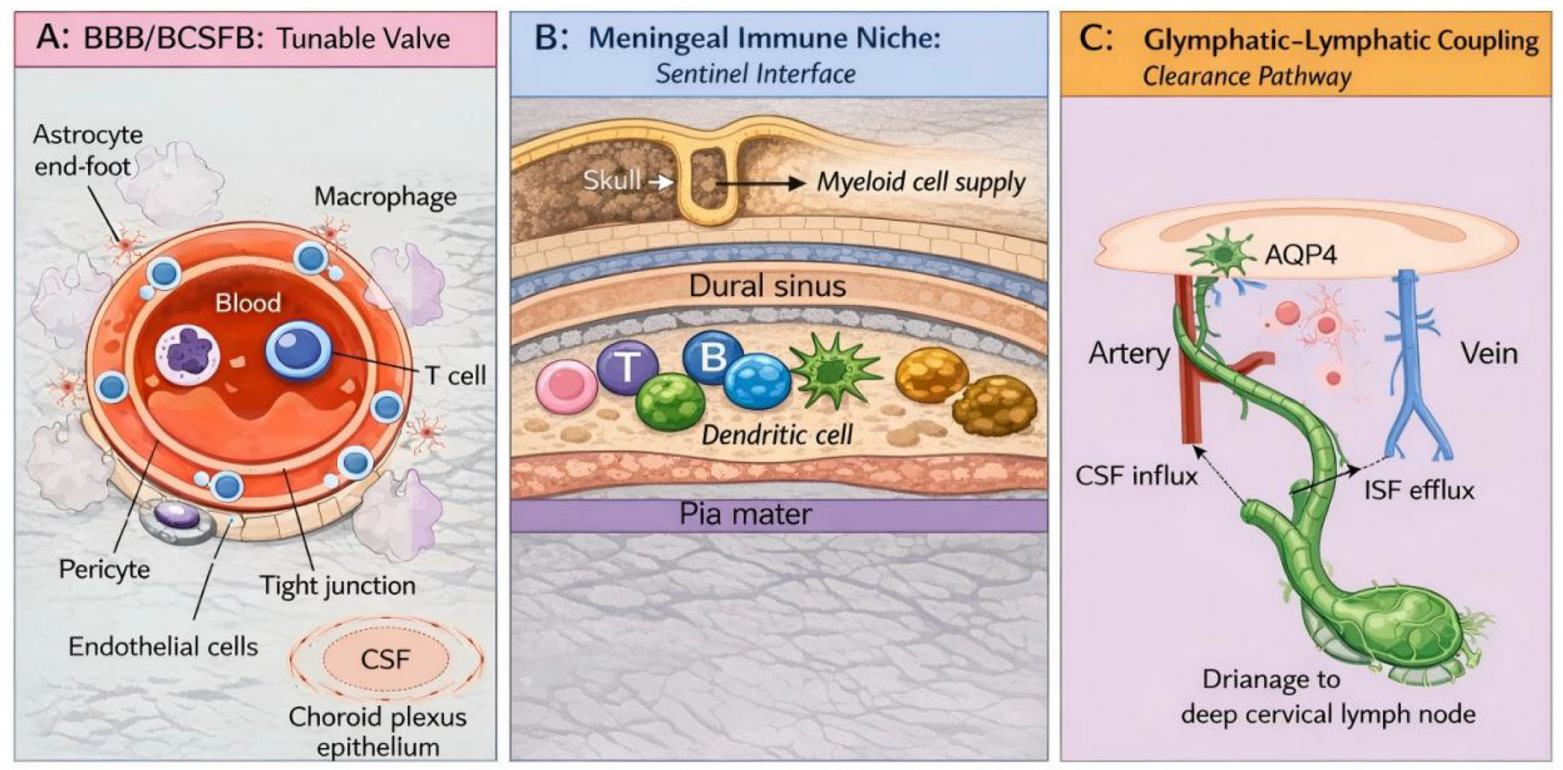
Structural portals for CNS immune modulation by neurosurgery. This schematic illustrates the three major anatomical interfaces through which neurosurgical interventions can influence CNS immune homeostasis. **(A)** The Blood-Brain Barrier (BBB)/Blood-Cerebrospinal Fluid Barrier (BCSFB), composed of endothelial cells, pericytes, and astrocytic end-feet, acts as a tunable valve regulating the entry of immune cells and therapeutics. Its integrity is dynamically altered in disease and by perioperative factors. **(B)** The Meningeal Immune Niche, located within the dura mater, harbors a diverse population of innate and adaptive immune cells (e.g., macrophages, T cells) that serve as sentinels, sensing CNS damage and orchestrating immune responses. This niche is directly affected by dural opening and repair. **(C)** Clearance Pathways, comprising the glymphatic system (for parenchymal waste clearance via perivascular channels) and meningeal lymphatic vessels (for draining solutes and immune cells to deep cervical lymph nodes), determine the resolution of inflammation. Their function is modifiable by CSF drainage, patient positioning, and sedation. Key neurosurgery-modifiable nodes are indicated for each portal.

Importantly, this clearance machinery is not merely a background physiological process but a modifiable therapeutic target embedded in routine neurosurgical practice. External ventricular or lumbar drainage directly alters CSF dynamics and may enhance the efflux of inflammatory mediators and blood breakdown products (40). Patient positioning, decompressive craniectomy, and dural repair techniques can influence intracranial pressure gradients, perivascular flow, and local meningeal immune responses, thereby modulating glymphatic and lymphatic function (41). Even anesthetic agents and sedation depth may impact clearance efficiency through effects on autonomic tone, sleep-like states, and neuroinflammation (42). Collectively, these modifiable factors reposition the neurosurgeon from a passive observer of immune outcomes to an active engineer of the CNS clearance landscape (**Figure 1**).

## 4 Immune dynamics across time windows: acute peak control, subacute resolution, and chronic plasticity limitation

### 4.1 Acute phase innate immunity: the “upper limit determiner” of secondary injury and edema

This section clarifies that the goal of acute immune management is not to eliminate inflammation, but to identify and “control the peak” of key inflammatory axes (e.g., the NLRP3 inflammasome), preventing their runaway amplification of secondary injury and cerebral edema, while preserving a window for subsequent resolution and repair.

The earliest immune events in acute brain injury are typically triggered by microglia, resident macrophages, and endothelial cells. DAMPs, blood degradation products, and nucleic acids/proteins from necrotic cells rapidly activate pattern recognition receptors (PRRs) and inflammatory cascades, inducing the release of cytokines and chemokines and recruiting peripheral neutrophils and monocytes (43). The key in this phase is not whether inflammation occurs, but whether it rapidly spirals out of control, potentially causing a ‘second hit’: worsened BBB damage, expanding edema, microcirculatory obstruction, and excitotoxicity, thereby may amplify the initial structural injury (44, 45).

At the molecular level, acute sterile inflammation is often driven by the PRR-inflammasome axis. The NLRP3 inflammasome, for instance, can be activated by extracellular ATP, ion dyshomeostasis, mitochondrial damage, and blood degradation products, thereby contributing to caspase-1-mediated maturation and release of IL-1β/IL-18 in experimental models (46, 47). This further amplifies vascular permeability, leukocyte adhesion/migration, and altered neuronal excitability (46, 48). Parallel pro-inflammatory networks include cytokine cascades like TNF-α and IL-6, which are thought to collectively contribute to edema and secondary injury through endothelial activation, glial polarization, and synaptic excitability modulation, although direct human evidence remains correlative (43, 49). Therefore, “peak control” is not merely about reducing cell numbers, but about limiting the sustained high expression of key pro-inflammatory axes (e.g., NLRP3-IL-1β-TNF-α) during the wrong time window, thereby preserving space for subsequent resolution and repair.

The common clinical observation of “similar hematoma volumes leading to vastly different edema and clinical worsening” likely relates to the intensity of the acute immune peak. Neutrophils release proteases, reactive oxygen species, and neutrophil extracellular traps (NETs), potentially causing endothelial injury and microthrombi. Monocytes/macrophages can exhibit either pro-phagocytic/clearance or persistently pro-inflammatory phenotypes depending on polarization. Microglia can protectively phagocytose debris but may also lower seizure thresholds by releasing pro-inflammatory mediators (50–52). Thus, the acute-phase objective resembles “peak control” rather than “complete suppression”: avoiding an excessively high and prolonged inflammatory peak to preserve the potential for subsequent resolution and repair (18).

### 4.2 Subacute phase resolution and clearance: determining recovery speed and chronic tailing risk

As the subacute phase begins, the inflammatory system should transition from “combat mode” to “mop-up mode”: clearing necrotic tissue and myelin debris, promoting vascular stabilization and barrier repair, and initiating remyelination and synaptic plasticity (53, 54). Key variables in this stage include macrophage/microglial phenotype switching, pro-resolving lipid mediators (SPMs), anti-inflammatory cytokines, and phagocytic clearance capacity (55, 56). Successful resolution leads to gradual edema resolution, restored neural network plasticity, and improved rehabilitation efficiency. Failed resolution, characterized by persistent inflammatory mediators, incomplete debris clearance, and sustained glial activation, pushes patients toward long-term symptom “tailing.”

Neurosurgical procedures significantly impact this phase. Postoperative cavity exudate and blood degradation products, if not cleared efficiently, can sustain inflammation. CSF drainage and shunting alter inflammatory mediator efflux. Dural repair and adhesion processes can modify the local immune niche. Superimposed infection or sterile inflammation can overwhelm the resolution system (18, 36, 57). Consequently, the subacute phase should become the second key window for neurosurgical immuno-translation. Rather than focusing all effort on forcefully suppressing acute inflammation, part of the strategy should shift toward promoting clearance and resolution to reduce long-term sequelae (53, 58).

### 4.3 Chronic phase immune remodeling: an immunological explanation for network reprogramming and epileptogenesis

Chronic dysfunction is often attributed to “irreversible neuronal loss.” However, growing evidence suggests a significant portion of chronic symptoms arises from abnormal neural network connection reprogramming, synaptic rearrangement, insufficient remyelination, and solidified glial scarring (59, 60). These are not purely neurobiological events; the immune system is deeply involved. Complement-mediated synaptic tagging and phagocytic pruning, persistent microglial activation, and long-term low-grade adaptive immune activation can shift network plasticity toward unfavorable directions, manifesting as attention deficits, executive dysfunction, mood disorders, sleep disturbances, or increased seizure risk (61, 62).

**Figure 2:**
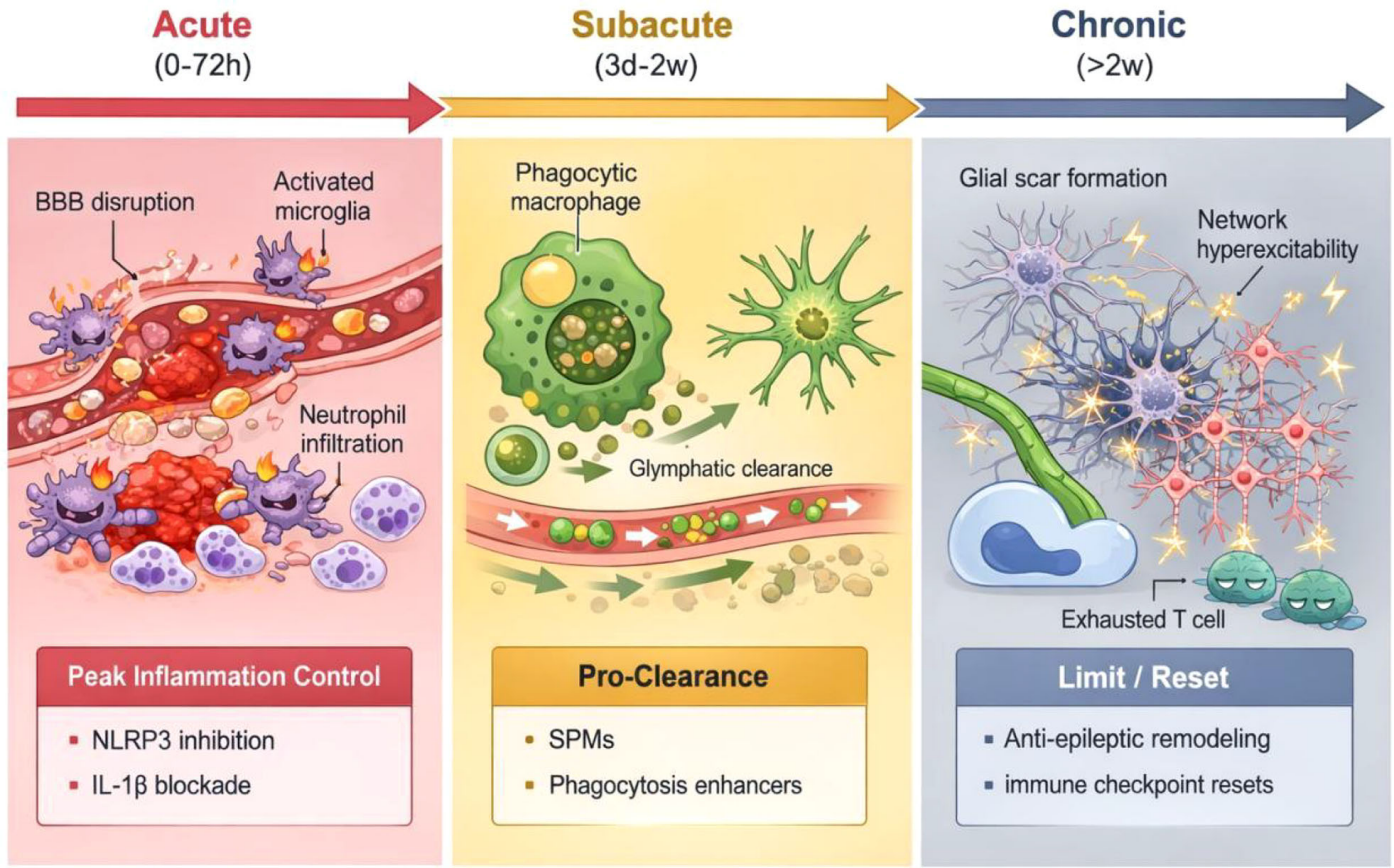
Temporal Dynamics and Staged Intervention Strategies Following a Neurosurgical Event. The three phases are presented from left to right without individual panel labels; they correspond to **(A)**, **(B)**, and **(C)** as described below. The graph depicts the conceptual trajectory of the neuroinflammatory response anchored to a surgical or injury event (e.g., resection, hemorrhage, trauma). The response is divided into three phases, each with a dominant pathological process and a corresponding strategic goal for immune intervention. **(A)** Acute Phase (Peak Control): A rapid innate immune response driven by DAMPs and the NLRP3 inflammasome can amplify secondary injury and cerebral edema. The goal is to control the peak of key pro-inflammatory axes without completely ablating inflammation. **(B)** Subacute Phase (Pro- Clearance & Resolution): The immune system should transition to debris clearance and tissue repair. Efficient phagocytosis and the action of pro-resolving mediators are critical. Failure here leads to prolonged inflammation. The goal is to promote resolution and prevent chronic 'tailing' of symptoms. **(C)** Chronic Phase (Limit/Reset): Persistent immune-glia interactions can drive pathological synaptic remodeling, network dysfunction (e.g., epileptogenesis), and, in tumors, a solidified immunosuppressive microenvironment. The goal is to limit maladaptive plasticity and reset the immune landscape to a state conducive to repair or therapeutic efficacy.

In neuro-oncology, the chronic phase also manifests as the solidification of an immunosuppressive tumor microenvironment (TME) and the establishment of tolerance: deepened T cell exhaustion, sustained myeloid-derived suppressor cell (MDSC) activity, and insufficient antigen presentation, hindering the establishment of durable effective memory responses to immunotherapy (21, 63). Therefore, chronic-phase strategies emphasize “plasticity limitation” and “reset”: limiting the network consolidation caused by deleterious immune-glia interactions, while simultaneously attempting to reset the TME via local immunoengineering or combinatorial therapies (**Figure 2**).

## 5 Immunological mechanisms in acute neurosurgical diseases: from hemorrhage-driven inflammation to “immunogenic microcirculatory dysfunction”

### 5.1 Hemorrhagic brain injury: sustained inflammation driven by blood degradation products and clearance bottlenecks

A hallmark of immune pathology in intracerebral hemorrhage (ICH) and subarachnoid hemorrhage (SAH) is the persistent stimulation of the innate immune system by blood components entering the brain parenchyma/subarachnoid space. Hemoglobin and its degradation products, iron dysmetabolism, and oxidative stress are recognized as key drivers in preclinical and associative human studies (64, 65). These blood breakdown products not only induce a pro-inflammatory response in microglia and macrophages but also damage endothelium, worsen microcirculation, and increase BBB permeability, thereby contributing to progressive cerebral edema in experimental settings (43, 66). The clinical observation that “edema does not strictly correlate linearly with hemorrhage volume” suggests that, beyond the initial bleed burden, the intensity of the immune peak and the efficiency of clearance jointly determine the edema trajectory and the ceiling for secondary injury (67).

In SAH, blood and inflammatory mediators in the subarachnoid space disrupt CSF circulation and meningeal lymphatic-glymphatic clearance pathways. This disruption couples with meningeal immune responses and alterations in vascular reactivity, ultimately linking to vasospasm, delayed cerebral ischemia, and long-term cognitive outcomes (68, 69). Therefore, the immunotranslation strategy for hemorrhagic brain injury should evolve from a simple “anti-inflammatory” approach to a closed-loop intervention: promoting the clearance of blood degradation products/inflammatory mediators, reducing endothelial inflammation and microcirculatory dysfunction, and facilitating the switch to a pro-resolution/repair phenotype (70).

It is worth noting that while sterile inflammation is clearly detrimental in the acute phase, complete abrogation of the immune response may impair hematoma resolution and tissue repair (71). This underscores the need for precisely timed, rather than broadly suppressive, immunomodulatory strategies.

### 5.2 Ischemia-trauma shared mechanism: immunogenic microcirculatory dysfunction leading to secondary injury and window shifts

While traumatic brain injury (TBI) and ischemia-reperfusion injury have different initiators (mechanical disruption vs. perfusion interruption/restoration), their immunopathology converges on a common “amplifier”: immunogenic microcirculatory dysfunction (45, 72, 73). Peripheral neutrophil and monocyte infiltration, complement activation, upregulated endothelial adhesion molecules, and microthrombosis are thought to create a vicious cycle, potentially further damaging potentially salvageable penumbral tissue. In reperfusion scenarios, oxidative stress and leukocyte adhesion are believed to drive the inflammatory cascade, contributing to ‘no-reflow’ phenomenon and microcirculatory perfusion failure, which may in turn lead to infarct expansion and edema exacerbation based on preclinical evidence (74, 75). In other words, the clinical scenario of “neurological worsening despite restored vascular patency on imaging” can often be explained within this coupled network of immune-endothelial-coagulation interactions.

This shared mechanism is also strongly influenced by the “systemic immune background.” TBI patients often exhibit a state of concurrent central inflammation and peripheral immunosuppression, increasing infection risk. Conversely, infections, fever, and inflammatory marker fluctuations can, in a feedback loop, amplify intracranial inflammation and edema (72, 76). Therefore, infection and inflammation management in the neurosurgical ICU should not be viewed merely as complication control but as an integral part of modulating the acute-phase immune trajectory. (For the chronic sequelae related to immune-synaptic mechanisms, refer to Section 3.3.).

From a translational strategy perspective, the key insight from this field is: single-target approaches often fail to address the complex cascade. A more feasible direction is time-window-oriented combinatorial management: focusing on reducing endothelial inflammation and microthrombotic tendency to limit immune peak amplification in the acute phase; promoting phagocytic clearance and inflammatory resolution in the subacute phase; and supporting repair and network plasticity in the chronic phase to reduce long-term functional deficits and epileptogenesis risk.

The acute sterile inflammation following hemorrhage or ischemia contrasts sharply with the chronic, smoldering immunosuppression characteristic of the GBM microenvironment. While acute injury triggers a rapid innate immune response aimed at debris clearance, the tumor ecosystem actively reshapes myeloid cells toward a suppressive phenotype that blunts adaptive immunity. Understanding this dichotomy is essential for designing phase-appropriate immunomodulatory strategies.

## 6 Brain tumor immune microenvironment: turning “resection” into the starting line for immunotherapy

### 6.1 Reshaping the battlefield: the myeloid-dominated immunosuppressive ecology of GBM and deconstructive logic

The frequent failure of immunotherapy in glioblastoma (GBM) often stems from “unfavorable battlefield conditions.” The immune cell composition in GBM is typically dominated by tumor-associated macrophages/microglia (TAMs), which frequently exhibit an M2-like/suppressive polarization in both preclinical models and human samples (77). Tumor-derived signals such as CSF-1/CSF-1R, IL-10, and TGF-β promote this inhibitory programming. Mechanisms like PD-L1 expression and metabolic suppression (e.g., via Arg1) further impair T cell infiltration, proliferation, and cytotoxicity (78). Coupled with hypoxia, abnormal vasculature, and profound spatial heterogeneity, this leads to a pattern of “local efficacy, systemic failure” (79). A more logical approach, therefore, may be to first remodel the myeloid-metabolic-vascular ecology, then address T cell checkpoints (‘release the brakes’), and guide combinatorial strategies using spatial stratification —a hypothesis that warrants prospective testing.

Despite the scientific rationale for targeting the immunosuppressive GBM microenvironment, immunotherapies have consistently failed to improve outcomes in phase 3 clinical trials. The CheckMate-143 trial comparing nivolumab to bevacizumab in recurrent GBM did not meet its primary endpoint of overall survival (mOS: 9.8 vs. 10.0 months; HR, 1.04) (80). Similarly, CheckMate-498 (MGMT-unmethylated newly diagnosed GBM) and CheckMate-548 (MGMT-methylated newly diagnosed GBM) both failed to show survival benefit with nivolumab added to radiotherapy/temozolomide, with CheckMate-548 even showing a trend toward harm (mOS: 28.9 vs. 32.1 months; HR, 1.10) (81, 82). These failures underscore that the challenges extend well beyond the well-characterized myeloid dominance.

Several interrelated factors likely explain this translational gap. First, GBM’s low mutational burden, sparse T cell infiltration, and lack of tertiary lymphoid structures render it immunologically “cold,” making checkpoint inhibition alone insufficient (80). Second, profound intratumoral heterogeneity—with regional variations in neoantigen expression and HLA loss—enables immune escape and outgrowth of antigen-negative clones (81). Third, the blood-brain barrier and perivascular niche create pharmacological sanctuaries, limiting antibody penetration and immune effector access (81). Fourth, immunotherapy-related neurotoxicity (cerebral edema, seizures) has necessitated steroid use that may counteract anti-tumor immunity (80). These observations suggest that future trials must move beyond binary questions toward spatially informed strategies: selecting patients based on immune phenotype, optimizing local delivery routes, and sequencing combinations to first “heat up” the tumor.

### 6.2 The immunological significance of surgery: a window of antigen release and a window of perioperative suppression risk

Surgical resection releases tumor antigens and DAMPs, theoretically enhancing antigen presentation and providing a foundation for subsequent “ignition strategies” like radiotherapy, oncolytic viruses, or vaccines. However, surgical stress, potential infections, and the use of corticosteroids can suppress the immune baseline, potentially offsetting these synergistic effects (83, 84). Thus, a key neurosurgical contribution lies in designing the “surgical event” as a programmable immune anchor point: implementing preoperative stratification, followed by intraoperative multi-regional sampling, and then postoperative window local delivery or combinatorial therapy, while using perioperative immune management to reduce confounding noise and toxicity. Current evidence is primarily from mechanistic studies and early-phase clinical trials; randomized controlled trials with standardized perioperative variable control remain a critical gap.

### 6.3 Aligning neurosurgical synergy with immunotherapy failure points

Translating “failure mechanisms” into “actionable synergy,” neurosurgical collaboration can prioritize addressing three high-frequency bottlenecks:

- Inadequate Target Exposure/Coverage (Drugs or effector cells fail to achieve sufficient spatial coverage within the heterogeneous tumor) → Utilize the postoperative window for local delivery into the resection cavity, controlled-release biomaterial platforms, or intrathecal/intraventricular administration to enhance target site exposure (85);
- Myeloid Suppression & Metabolic/Vascular Barriers (TAM dominance, hypoxia, and abnormal vasculature causing “local efficacy, systemic failure”) → First employ myeloid-metabolic-vascular reprogramming strategies to improve battlefield conditions, then sequence with ICIs (“T cell brake release”) or other therapies, guided by spatial stratification (86);
- Neurotoxicity & Perioperative Risks (Edema, seizures, infection/corticosteroid use negating immune synergy) → Through perioperative immune management, dose escalation with monitoring, and efforts to “confine responses to the target area while controlling systemic burden,” enhance treatment tolerability and interpretability (87).

## 7 Perioperative immune management: the most actionable translational lever in neurosurgery

### 7.1 Surgical stress, anesthesia, and analgesia: the “invisible switch” of the immune baseline

(1) Stress Axis and Sympathetic Activation Drive Short-Term Immunosuppression and Inflammation Redistribution.

Surgical trauma, pain, and sleep deprivation activate the sympathetic-adrenal-medullary (SAM) axis and hypothalamic-pituitary-adrenal (HPA) axis. This alters the distribution of peripheral immune cells and cytokine profiles, typically manifesting as a mixed state of “enhanced local inflammation coupled with depressed systemic immune function” (10, 88, 89). In oncology patients, this state may impair anti-tumor immunity; in acute brain injury patients, it may increase the risk of infection and secondary damage.

Different anesthetic agents, analgesic regimens, and sedation depths can influence inflammatory markers and immune cell activity. Clinically, the key is not identifying a single “best drug,” but rather incorporating anesthesia and analgesia as documented, standardized variables into study designs to reduce noise in immune trials. Furthermore, perioperative medications are not merely correlative variables; they may directly impinge upon effector immune pathways. For instance, neutrophil extracellular traps (NETs) are associated with microthrombosis, endothelial damage, and barrier disruption, serving as a significant amplifier in acute brain injury and perioperative microcirculatory dysfunction. Certain anesthetics/sedatives may influence NETosis propensity and inflammatory thresholds via effects on the stress axis, oxidative stress, and epigenetic regulation (90–92). Additionally, histone deacetylases (HDACs) are involved in immune gene transcription and the programming of inflammatory responses. The indirect impact of some perioperative drugs on HDAC-related pathways could cause substantial differences across the ‘peak control, resolution, and repair’ timeline (93). Therefore, in future CNS immune trials, anesthetic and analgesic strategies should not only be recorded but also be considered as optimizable “immune covariates.” Consequently, anesthesia protocols and sedation depth should be prospectively defined as covariates/stratification factors in CNS immune study designs.

### 7.2 Corticosteroids: the essential need for edema control vs. the core contradiction for immunotherapy

(1) The Main Issue is Not “Whether to Use,” but “Dose and Timing Determine Benefit vs. Harm”.

Dexamethasone and similar agents are widely used and generally effective for controlling vascular permeability and edema. However, they can suppress T cell responses, impact antigen presentation, and alter the TME. A more rational approach is: “minimum effective dose + shortest necessary duration + avoiding high doses during critical immune-activation windows” (94, 95).

(2) Decision-Making Should Evolve from “Habit” to “Mechanism”: Balancing Edema Risk vs. Immunotherapeutic Benefit.

When combining immunotherapy in brain tumor patients, it is essential to define: what imaging/symptoms necessitate steroids; when can doses be tapered; which therapies (e.g., vaccines, oncolytic viruses, checkpoint inhibitors) are more sensitive to steroids and require stricter control (96, 97).

(3) Exploring Alternative Strategies to Reduce Steroid Dependence: Creating Favorable Conditions for Immunotherapy.

This includes more precise local delivery methods, strategies to improve microcirculation and barrier management, and integrated approaches targeting permeability and vascular abnormalities. Even if evidence is still evolving, proposing testable directions adds translational value.

### 7.3 Infection and inflammation: not merely complications, but events that reshape the immune ecology

(1) Infections Alter Myeloid Lineages and Systemic Inflammatory Load, Reciprocally Affecting Cerebral Edema and the BBB.

Postoperative infections often cause systemic inflammatory fluctuations, increasing BBB permeability and amplifying intracranial inflammation, thereby inducing or exacerbating edema, seizures, and altered consciousness (98, 99).

(2) Antibiotic Therapy and Inflammation Control Must Be Incorporated into the “Background Therapy Standardization” of Immune Trials.

Many immunotherapy studies fail not because the strategy is ineffective, but because perioperative infections, antibiotic use, and inflammatory fluctuations introduce significant noise. Systematically recording, standardizing, and incorporating these factors into inclusion/exclusion criteria or stratification is key to improving reproducibility (100, 101).

(3) Sterile Inflammation is Equally Important: Postoperative Fever, CSF Pleocytosis, and Material Reactions.

Originating from biomaterials, blood degradation products, and meningeal reactions, these can cause fever, increased CSF cell counts, and edema. For immune-related clinical research, systematically differentiating, coding, and stratifying these events from true infections is crucial for enhancing data interpretability and reproducibility (102, 103).

## 8 Immunotherapy and local immunoengineering: delivery and timing determine success or failure

### 8.1 Immune checkpoint inhibition: single-agent limitations and the key of “ignition before releasing the brakes”

(1) Why Single-Agent Therapy is Often Weak: Insufficient Baseline and Suppression Beyond PD-1/CTLA-4.

In CNS tumors and chronic inflammatory conditions, the microenvironment is often characterized by T cell scarcity, strong myeloid suppression, and insufficient antigen presentation drive. Merely blocking PD-1/CTLA-4 often means ‘releasing brakes with no engine power,’ as suggested by the limited efficacy of single-agent ICIs in GBM trials (104). Furthermore, alternative inhibitory receptors such as LAG-3, TIGIT, and TIM-3 can sustain the exhausted T cell phenotype, limiting efficacy (105).

(2) A More Rational Approach: Prioritize Combinations, Neurosurgery Focuses on Concentrating Responses in the Target Area.

A more viable strategy is “first enhance antigen presentation and T cell infiltration (ignition), then reverse exhaustion (release brakes),” guided by spatial stratification/immune phenotyping for combination selection. The neurosurgical synergy lies in leveraging the postoperative window and local delivery routes (surgical cavity, intrathecal, intraventricular) to increase target site drug/immune cell exposure, while employing perioperative management to reduce systemic toxicity (106, 107).

### 8.2 Vaccines and oncolytic viruses: “ignition” platforms for the postoperative window

(1) Advantages and Mechanisms: Activating Type I Interferon Programs, Enhancing Antigen Presentation.

Vaccines and oncolytic viruses can promote antigen presentation and immune memory formation during the postoperative window. Mechanistically, they often trigger Type I interferon responses via pathways like cGAS–STING or RLR–MAVS, creating a favorable immune foundation for subsequent checkpoint blockade or cell therapy (108, 109).

(2) Major Bottlenecks: Edema/Seizure Risk and Inadequate Spatial Coverage.

Local inflammatory initiation can carry risks of edema and seizures. Furthermore, tumor spatial heterogeneity leads to uneven therapeutic coverage. Practical solutions rely on dose escalation, combination with anti-edema agents, and more controllable local delivery methods (23, 110).

### 8.3 Cell therapy and bispecific agents: success depends on sustained function after entry

(1) Challenges: Counteracting Suppression, Preventing Exhaustion, and Managing Neurotoxicity.

Entry into the CNS is just the first step. The greater challenge is maintaining effector function within the immunosuppressive microenvironment while avoiding exhaustion. Concurrently, cerebral edema, seizures, and other neurotoxicities must be core components of risk management.

(2) Neurosurgical Advantage: Local Administration + Stratified Selection to Enhance Reproducibility.

Intrathecal, intraventricular, or intracavitary administration can increase target site exposure and reduce systemic toxicity, but requires strict monitoring and stepwise dosing. Combining this with spatial stratification to select patients, targets, and combinations holds promise for improving efficacy and safety margins (14, 111). CNS immune enhancement strategies should predefine a three-tiered safety framework: stratified enrollment, early intensive monitoring, and delivery/dose escalation protocols. This aims to improve reproducibility and better dissociate efficacy from toxicity. Current evidence remains primarily feasibility/safety and early signal data; efficacy gains depend on prospective studies optimizing stratification and combinations (106, 112).

## 9 Biomarkers and omics stratification: transforming neurosurgical sampling advantage into a “reproducible system”

### 9.1 Tissue–CSF–peripheral blood: a stratification framework for within-subject triangulation

(1) Tissue Answers “Spatial Heterogeneity and Niche,” CSF Answers “CNS Dynamics,” Peripheral Blood Answers “Systemic Context”.

Relying on a single source can be misleading: peripheral blood does not equal the brain interior, and a tissue snapshot does not capture dynamic processes. Integrating all three significantly enhances explanatory power and follow-up capability (113–115).

(2) Timing is Equally Critical: Pre-Intra-Postoperative Windows Determine Biomarker Significance.

Perioperative immune states change rapidly. Defining standardized sampling timepoints is essential; otherwise, comparisons across studies are compromised. Proposing explicit standard time windows (e.g., ‘preoperative baseline, early postoperative (3–7 days), and mid-term (2–4 weeks)’) itself constitutes a methodological contribution (113, 116).

(3) Replacing “Disease-Specific Marker Lists” with “Immune-State Typing”.

Examples include: Barrier-Disrupted, Myeloid-Suppressive, T cell-Exhausted/Depleted, Clearance-Impaired, Resolution-Failure types. Once established, such typing can directly guide intervention selection (117).

(4) Clinical Implementation Can Start with a Minimum Indicator Set.

Peripheral blood (NLR, CRP/IL-6, lymphocyte counts), CSF (cell count/differential, protein/glucose, key cytokines like IL-6/IL-1β or surrogates), imaging (permeability/perfusion or edema volume changes), and perioperative variables (steroid dose, infection events, drainage strategy). Spatial omics can be layered on this foundation to refine typing and mechanistic understanding (118, 119).

### 9.2 Single-cell and spatial omics: key tools for explaining heterogeneity and treatment failures

(1) Single-Cell Reveals Key Subsets, Spatial Omics Reveals “Who Interacts with Whom”.

Immunosuppression in GBM is often dominated by a few key ecological niches (e.g., perivascular, hypoxic, infiltrative edge). Spatial interaction information can directly inform delivery and targeting strategies (120).

(2) From “Description” to “Prediction”: Turning Omics into Stratification and Efficacy Forecasting Tools.

**Figure 3:**
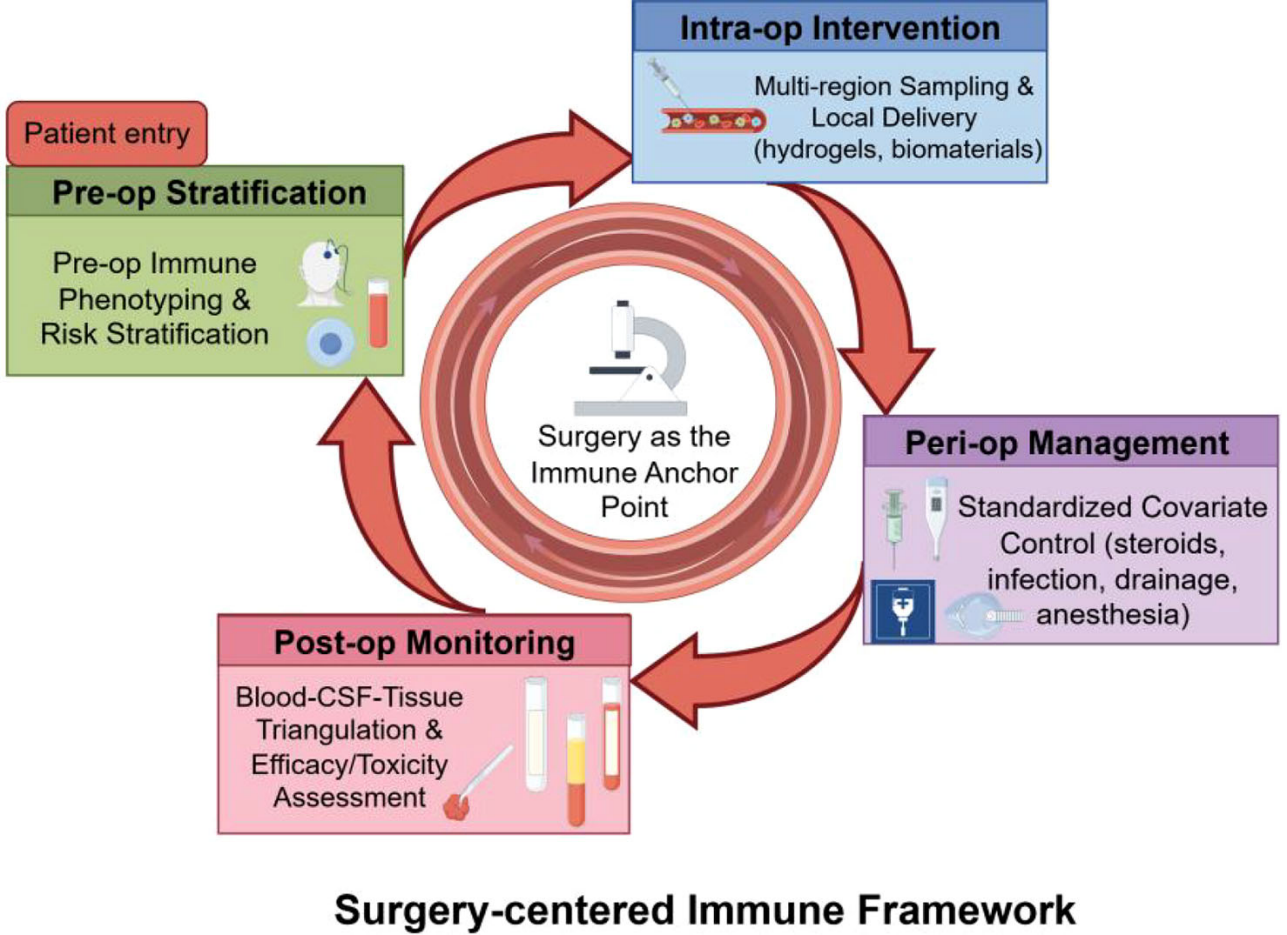
A closed-loop translational framework with surgery as the immune anchor point. This flowchart proposes a verifiable and iterable framework for integrating neuroimmunology into clinical research and practice. The surgical event serves as the central biological anchor point, organizing a four-stage loop: ① Preoperative Stratification: Patients are phenotyped based on immune state (e.g., barrier integrity, myeloid suppression) and risk using a minimum dataset (Table 1). ② Intraoperative Intervention & Sampling: Multi-regional tissue sampling maps spatial heterogeneity. This window is also used to deploy local immunoengineering strategies (e.g., controlled-release hydrogels) directly into the resection cavity. ③ Perioperative Standardized Management: Key covariates, including corticosteroid use, infection, drainage strategies, and anesthesia protocols, are meticulously recorded and standardized to reduce confounding noise. ④ Postoperative Dynamic Monitoring: Longitudinal sampling of blood and CSF, coupled with clinical and imaging follow-up, assesses efficacy, toxicity, and mechanistic biomarker changes. ⑤ Feedback & Iteration: Findings from monitoring feed back to refine stratification algorithms, optimize delivery methods, and guide combination therapies for subsequent patients or trials.

For example, using spatial maps to predict which patients are more likely to develop edema or neurotoxicity, or which are more likely to benefit from specific immunocombinations. These predictive models should be primarily built around the surgical anchor point: calibrated with intraoperative spatial sampling and postoperative dynamic follow-up to inversely optimize delivery routes, combination regimens, and perioperative management parameters (121, 122) (**Figure 3**).

## 10 Future roadmap: embedding neurosurgical interventions within the immunological closed loop

### 10.1 From disease categories to immune-state typing

Outcome variability within a single disease entity is often determined more by the underlying immune state than by conventional imaging or pathological labels alone. We propose adopting a four-dimensional immune-state classification—assessing barrier integrity, myeloid suppression, T cell repertoire/exhaustion, and clearance/resolution capacity—as a common language for both clinical practice and trial design. This classification can be operationalized using a “minimum indicator set + omics enhancement” approach for practical stratification (123, 124).

#### 10.1.1 Implementation challenges: from concept to clinical reality

While the proposed framework offers a coherent roadmap, its translation into practice faces several hurdles. First, the “minimum dataset” (**Table 1**) requires standardized collection of perioperative variables that are often inconsistently recorded across centers, raising feasibility concerns in resource-limited settings. Second, multi-region sampling and dynamic CSF monitoring, though scientifically powerful, introduce ethical and logistical considerations—patients must consent to additional procedures, and biobanking infrastructure must ensure proper sample processing and data integration in compliance with privacy regulations (125). Third, the financial burden of spatial omics and local delivery platforms is substantial; cost-effectiveness analyses and industry-academic partnerships will be essential for moving from early-phase trials to larger studies (126). Finally, the regulatory pathway for locally delivered immunotherapies remains undefined, requiring early engagement with regulatory agencies (127). Acknowledging these challenges does not diminish the framework’s promise; rather, it underscores the need for pragmatic, stepwise implementation—starting with feasibility studies in academic centers, establishing standardized protocols, and progressively validating the approach in broader populations.

### 10.2 From systemic delivery to local immunoengineering

A key bottleneck for CNS therapies is delivery and spatial heterogeneity. Neurosurgical routes—including the resection cavity, intrathecal, and intraventricular spaces—coupled with controlled-release platforms (e.g., hydrogels, biomaterials), offer a strategic breakthrough point. Crucially, local engineering must be intrinsically linked to robust risk management protocols for edema, seizures, and neurotoxicity, as well as optimized perioperative care. Only by deliberately separating efficacy signals from toxicity profiles can we enhance reproducibility and clinical adoption (23, 128).

### 10.3 From scattered metrics to an executable trial checklist

Future trial design should shift from enrolling patients based solely on pathological diagnosis to stratification based on immune phenotypes and risk profiles. The time axis should be firmly anchored to the “surgical event, with the perioperative period as the core intervention window,” standardizing sampling and follow-up schedules. Endpoints should adopt a tripartite structure integrating clinical efficacy, immune mechanism modulation, and safety, thereby forming a verifiable and iterable translational pathway (129, 130).

## 11 Conclusion

Neurosurgery is transitioning from an era of “structural treatment” to a new paradigm of “structural + immune systems engineering.” Discoveries related to the meningeal immune niche, meningeal lymphatic–glymphatic clearance pathways, and the dynamic regulation of CNS barriers have transformed CNS immunity from a “black box” into a comprehensible, measurable, and intervenable system. Neurosurgery’s unique advantages in tissue sampling, local therapeutic delivery, and perioperative management provide a practical foundation for establishing a closed-loop framework encompassing “structural pathways, immune dynamics, delivery/timing, and efficacy/toxicity.”

The key to enhancing the success rate of CNS immunotherapies in the future lies not in targeting single molecules in isolation, but in leveraging surgery as a biological anchor point. Through immune-state phenotyping and local immunoengineering, we can integrate mechanistic insights with clinical pathways into a verifiable and iterable translational system. This paradigm shift positions the neurosurgeon not only as a master of anatomy and technique but as a central architect of the patient’s post-injury or post-resection immune landscape, ultimately aiming to improve long-term neurological and oncological outcomes.
